# Fusidic Acid: A Neglected Risk Factor for Statin-Associated Myopathy

**DOI:** 10.1177/1179546818815162

**Published:** 2018-12-04

**Authors:** Josefine Rönnqvist, Pär Hallberg, Qun-Ying Yue, Mia Wadelius

**Affiliations:** 1 Uppsala University Hospital and Department of Medical Sciences, Clinical Pharmacology and Science for Life Laboratory, Uppsala University, Uppsala, Sweden; 2 Swedish Medical Products Agency, Uppsala, Sweden

**Keywords:** adverse drug reactions, cardiovascular drugs, drug-induced disorders, drug safety, dyslipidemia, hypercholesterolemia, hyperlipidemia, myopathy and statins

## Abstract

**Background:**

Statins are widely used lipid-lowering drugs used for the prevention of cardiovascular disease. Statins are known to cause myopathy, an adverse drug reaction with various clinical features rhabdomyolysis.

**Objective:**

To describe clinical characteristics of statin-treated individuals who experienced myopathy and identify risk factors of statin-associated myopathy.

**Methods:**

A retrospective study was conducted on cases of statin-associated myopathy reported to the Swedish Medical Products Agency. Clinical factors were compared between cases and statin-treated controls not diagnosed with myopathy. Statistical methods were univariate and multivariate logistic regression and results were presented as odds ratio (OR) with 95% confidence interval (CI). To correct for multiple comparisons, the cutoff for statistical significance was set to *P* < .0017.

**Results:**

In total, 47 cases of statin-associated myopathy were compared with 3871 treated controls. Rhabdomyolysis was diagnosed in 51% of the cases. Markers for cardiovascular disease were more common in cases than controls. Statistical analysis revealed the following independent risk factors for myopathy: high statin dose (OR = 1.54, calculated using the standard deviation 19.82, 95% CI = 1.32-1.80, *P* < .0001), and concomitant treatment with fusidic acid (OR = 1002, 95% CI = 54.55-18 410, *P* < .0001), cyclosporine (OR = 34.10, 95% CI = 4.43-262.45, *P* = .0007), and gemfibrozil (OR = 12.35, 95% CI = 2.38-64.10, *P* = .0028).

**Conclusions:**

The risk of myopathy increases with statin dose and cotreatment with cyclosporine and gemfibrozil. Concomitant fusidic acid has previously only been noted in a few case reports. Considering that use of fusidic acid may become more frequent, it is important to remind of this risk factor for statin-associated myopathy.

## Introduction

Statins, or hydroxy-methylglutaryl-coenzyme A reductase (HMGCR) inhibitors, substantially reduce cardiovascular morbidity and mortality in both primary and secondary prevention in men and women of all age groups.^1–3^ The importance of statin treatment is well-documented and the treatment indications are becoming wider. In 2016, 9% of the Swedish population was treated with statins and the use is increasing every year.^4^ The safety of statins is sometimes questioned as treatment is associated with an adverse drug reaction (ADR) affecting skeletal muscles, often defined as myopathy. Myopathy is a general term for muscle disease and refers in this study to a toxic, drug-induced myopathy with several clinical phenotypes, ranging from muscle symptoms such as pain and weakness to rhabdomyolysis with marked creatine kinase (CK) and myoglobin elevations and sometimes acute renal failure.^5^ The incidence of rhabdomyolysis is estimated to be as low as 3.4 per 100 000 person-years, but it can be life-threatening.^6^ Autoimmune necrotizing myopathy is another rare and serious form of myopathy with persisting or progressing of symptoms despite withdrawal of the statin.^5^

Factors repeatedly mentioned in the literature to increase the risk of statin-associated myopathy are a high statin dose, advanced age, female sex, low body mass index, interacting drugs, and concomitant diseases such as impaired renal or hepatic function, hypothyroidism, and diabetes.^7,8^ Individuals with a previous history or family history of myopathy are also at higher risk.^9^ Genetic predisposing factors have been identified for statin-associated myopathy, and the most significant research finding is a polymorphism in the solute carrier organic anion transporter family member 1B1 (SLCO1B1) gene.^10^ This gene variant is associated with decreased transport of statins into hepatocytes, in particular of simvastatin, and consequently higher blood and presumably higher skeletal muscle statin concentrations.^10^ This leads to an increased risk of statin-associated myopathy; however, the molecular mechanisms are as yet unclear. Preemptive testing of SLCO1B1 has not been implemented into clinical practice in Sweden, and the genetic contribution of other risk variants remains to be investigated.

It is of great importance to increase the knowledge about risk factors for statin-associated myopathy so that treatment for patients at higher risk can be modified. Although most muscle symptoms are not as severe as rhabdomyolysis, they still lead to poor quality of life, discontinuation of statin treatment, and subsequently a failure to prevent cardiovascular disease.

This study describes the clinical characteristics of statin-treated individuals who experienced myopathy and compares them with treated controls without myopathy with the aim to identify risk factors.

## Material and Methods

A retrospective case-control study was performed as part of the SWEDEGENE project, a collaboration between Uppsala University, the Swedish Medical Products Agency (MPA), and Karolinska Institutet that aims to find genetic causes of ADRs.^11^ SWEDEGENE has created a database consisting of clinical data and DNA samples from patients who have had different kinds of ADRs, including statin-associated myopathy.

### Patient recruitment and data collection

All cases of statin-associated myopathy reported to the MPA in Sweden between January 1, 1990, and December 6, 2016, were retrieved. The reporter of the ADR was asked whether the patients were considered suitable for the study. If so, the patients were contacted and those who agreed to participate received a study kit containing an informed consent form, a questionnaire, and material to acquire blood samples. Clinical data about demographics, medical history, allergies, smoking, and alcohol habits at time of onset and drug treatment during 3 months before the time of onset were obtained from a standardized questionnaire completed through a telephone interview. Copies of the patients’ medical records were obtained, including ADR diagnosis, lab data of CK, and myoglobin values in plasma and/or urine, and when available muscle biopsy results. The previously mentioned data together with information from the ADR report were entered into the SWEDEGENE database.

Statin-treated controls were retrieved from people in the Swedish Twin Register that were born 1911–1958.^12^ This register is linked to 2 registers run by the Swedish National Board of Health and Welfare. The controls’ prescribed and collected drugs between July 2005 and December 2014 were obtained from the National Prescribed Drug Register^13^ using Anatomical Therapeutic Chemical (ATC) Classification codes. This register started in July 2005, and thus the date of the first prescription was after this date even if the patient had been exposed to statins before then. Drugs taken 3 months before and after the first collected statin prescription were extracted. Over-the-counter (OTC) drugs are not included in the Prescribed Drug Register. *International Statistical Classification of Diseases and Related Health Problems* (ICD) diagnoses registered before the first statin prescription were obtained from the National Patient Register.^14^ This register includes inpatient care and outpatient visits but does not cover primary care on a national level, and has a low sensitivity for some diagnoses.^15^ The only demographical variables available for the controls were age and sex.

### Inclusion and exclusion criteria

The patient needed to be at least 18 years old and able to give informed consent to participate in the study. All included cases had had myopathy with different severities. A statin was the suspected drug in all cases and all experienced improvement on dechallenge. The diagnosis of myopathy was validated in every case using medical records, *International Classification of Diseases, Tenth Edition* (ICD-10) diagnosis, ADR reports, and lab data. Controls with only one statin prescription outtake were excluded as they might have stopped taking the drug due to muscle symptoms. In addition, controls with ICD-10 codes for myopathy or myositis registered up to 3 months after the first statin prescription were excluded.

### Data analysis

Data from the SWEDEGENE database and the Twin Register were exported to Excel spreadsheets for statistical analyses. Concomitant diseases and drugs that occurred in at least 15% of the cases were compared between cases and controls based on power calculations. Drugs were categorized in drug classes according to the ATC system. Inhaled drugs, eye drops, and OTC drugs such as dietary supplements, proton-pump inhibitors, and pain killers were not compared between cases and controls. Other suspected drugs in the ADR report, in addition to statins, were studied as candidate factors both for evaluation of the association with myopathy and for validation as some of them are known to interact with statins. The ICD diagnoses with estimated low sensitivity in the Twin Register were not compared between cases and controls. Statin doses were converted to simvastatin dose equivalents according to the dose conversion charts based on reduction in low-density lipoprotein cholesterol.^16,17^ Simvastatin equivalent dose = pravastatin dose divided by 2, fluvastatin dose divided by 4, atorvastatin dose times 2, and rosuvastatin dose times 8.

### Statistical analysis

With 47 cases and 3871 controls, we had 80% power to detect an odds ratio (OR) ⩾3.7 using factors that occur in at least 7 (15%) of the cases. This is based on a significance level of *P* < .05. Descriptive statistics were presented with median values with quartiles for numeric continuous variables or number and proportion for nominal variables. JMP 13.1.0 (SAS Institute Inc., Cary, NC, USA)^18^ was used for statistical calculations. Logistic regression was used and ORs with 95% confidence interval (CI) and *P* values were calculated in univariate models. Statistical comparisons were corrected for the number of tested variables (.05/29) giving a cutoff for statistical significance of *P* < .0017. Nominal significance was defined as *P* < .05. Statistically significant variables in the univariate analysis were included in a multivariate model, in which adjusted ORs with 95% CIs were calculated.

### Ethics

All cases and controls had given informed consent and the data were deidentified. The study was performed according to the ethical standards of the local ethics committee (Dnr 2008/213 and Dnr 2010/231, Uppsala, Sweden and Dnr 2007-644-31, Stockholm, Sweden), the Declaration of Helsinki, and the United Nation’s Universal Declaration of Human Rights.

## Results

In total, 107 cases of statin-associated myopathy had been reported to the MPA during the studied period. Of these cases, 60 were not possible to recruit for the following reasons: inability to reach the reporter or the patient (30), the patient had deceased (10), the reporter considered the patient unsuitable for the study (7), the patient chose not to participate (9), the patient did not comply with the study procedure (1), and the ADR report was a duplicate (3). The remaining 47 cases were included. A total of 4090 statin-treated controls were extracted from the Twin Register. In all, 15 of these controls had been diagnosed with myopathy and were excluded. Another 204 controls were excluded as they had only one statin prescription. In all, 3871 controls were included. The case group was heterogeneous. All cases, except one, reported muscle symptoms and the most frequently reported symptom was muscle pain in combination with weakness. All cases with available lab data had elevated CK and/or myoglobin values in different degrees. Most of the cases, 79%, had marked CK elevations above 10 times the upper limit of normal (ULN), suggesting a significant muscle breakdown (Table 1). The 2 cases with low CK elevations (1.2 and 2× ULN) had myoglobin elevations of 15 and 1057× ULN, respectively. Five cases with overwhelming evidence of myopathy according to the medical record or physician’s report were included despite no available CK values. These cases had according to the reporting physician: (1) elevated myoglobin and CK, (2) severe myopathy, (3) a muscle biopsy showing necrotizing myopathy positive for HMGCR antibodies, (4) rhabdomyolysis with acute renal impairment and elevated liver enzymes, and (5) rhabdomyolysis with acute renal impairment and elevated myoglobin according to the physician’s report.

**Table 1.** Clinical characteristics of patients experiencing statin-associated myopathy.

Variables	Cases (n = 47)		
Demographic variables			
Age, y	*60*	66	*72*
Body mass index, kg/m^2^	*24*	27	*31*
Weekly alcohol consumption, standard drink	*0*	1	*2* ^a^
Sex, male	29	62%	
Ethnicity, Swedish	42	89%	
Smoking	5	11%	
Treatment indication			
Hyperlipidemia	8	17%	
Familiar hypercholesterolemia	2	4%	
Ischemic heart disease	4	9%	
Unknown	33	70%	
Time of treatment before onset, d	*81*	700	*1825* ^b^
ADR characteristics			
Muscle symptoms without CK or myoglobin data	2	4%	
Muscle symptoms + CK or myoglobin elevations	20	43%	
Rhabdomyolysis	24	51%	
Autoimmune necrotizing myopathy	1	2%	
Reported symptoms			
Muscle pain and weakness	21	45%	
Muscle pain, weakness, and dark urine	15	32%	
Muscle pain	3	6%	
Muscle weakness	6	13%	
Muscle cramps	1	2%	
No reported symptoms	1	2%	
Lab data			
Elevated CK value <4× ULN	2	4%	
Elevated CK value 4-10× ULN	3	6%	
Elevated CK value >10× ULN	37	79%	
No CK values available	5	11%	
Elevated myoglobin in plasma or urine	31	66%	
No myoglobin values available	16	34%	
ICD-10 diagnosis in medical records			
G72.0 Drug-induced myopathy	14	30%		
G72.8 Other specified myopathies	12	26%	
G72.9 Myopathy, unspecified	1	2%	
M60.8 Other myositis	1	2%	
M60.9 Myositis, unspecified	1	2%	
M62.8 Other specified disorders of muscle	1	2%	
M79.1 Myalgia	1	2%	
T88.7 Unspecified adverse effect of drug or medicament	1	2%	
No code available	15	32%	

Abbreviations: CK, creatine kinase; ULN, upper limit of normal.

Median values of continuous variables are presented with the lower quartile and the upper quartile in italics. Percentages are calculated of from total number of cases, excluding cases with missing values.

a Data lacking for 2 cases.

b Data lacking for 10 cases.

In total, rhabdomyolysis was diagnosed in 51% of the cases according to the reports.

No significant differences were seen in age or sex between the groups (Table 2). The cases tended to be overweight (median body mass index = 27 kg/m^2^). About 11% of the cases had ancestry outside Sweden including Sweden + Norway (1), Sweden + Germany (1), Serbia + Austria (1), Finland (1), and Bangladesh (1). Median time from initiation of statin treatment to ADR onset was 700 days but ranged widely from a minimum of 3 to maximum 6950 days. Total time of treatment was not available for controls, but the median time between the first and the latest prescription in the interval 2005 to 2014 was 5 years (quartile 1 = 2, quartile 3 = 8).

**Table 2.** Univariate logistic regression of the association between statin myopathy and clinical factors.

Variable	Cases (n = 47)	Controls (n = 3871)	OR	95% CI	*P* value
Age	*60* 66 *72*	*62* 67 *73*	0.97	0.93–1.00	.0813
Statin dose^a^	*20* 40 *80*	20 20 40	1.64	1.45–1.86	**<.0001** *
Sex, male	29 (62%)	2072 (54%)	1.40	0.77–2.53	.2661
Angina pectoris or myocardial infarction	22 (47%)	811 (21%)	3.32	1.86–5.92	**<.0001** *
Stroke incl. transient ischemic attack	10 (21 %)	481 (12%)	1.90	0.94–3.86	.0732
Atrial fibrillation	7 (15%)	209 (5%)	3.07	1.36–6.93	.**0070**
A10A Insulins and analogues	7 (15%)	227 (6%)	2.81	1.24–6.34	.**0129**
A10B Blood glucose–lowering drugs excl. insulins	8 (17%)	470 (12%)	1.48	0.69–3.20	.3127
B01AA Vitamin K antagonists	10 (21%)	254 (7%)	3.85	1.89–7.83	.0002*
B01AC Platelet aggregation inhibitors excl heparin	27 (57%)	1896 (49%)	1.41	0.79–2.52	.2506
C01DA Organic nitrates	7 (15%)	621 (16%)	0.92	0.41–2.05	.8311
C03A Thiazides	8 (17%)	811 (21%)	0.77	0.36–1.66	.5113
C03C Loop diuretics	14 (30%)	360 (9%)	4.14	2.19–7.80	**<.0001** *
C03D Potassium-sparing agents	7 (15%)	305 (8%)	2.05	0.91–4.61	.0838
C07A Beta-blocking agents	30 (64%)	1663 (43%)	2.57	1.40–4.72	.**0023**
C08CA Dihydropyridine derivatives	8 (17%)	746 (19%)	0.86	0.40–1.85	.6976
C09A ACE-inhibitors	16 (34%)	1034 (27%)	1.42	0.77–2.60	.2617
C09C Angiotensin II antagonists	11 (23%)	486 (13%)	2.13	1.08–4.21	.**0299**
Suspected drugs other than statins ^b^					
Cyclosporine	3 (6%)	4 (0.1%)	65.91	14.33–303.24	**<.0001** *
Fusidic acid^c^	5 (11%)	0 (0%)	NA	NA	NA
Allopurinol	5 (11%)	70 (1.8%)	6.46	2.48–16.83	.0001*
Ticagrelor	3 (6%)	29 (0.75%)	9.03	2.65–30.76	.0004*
Clindamycin	3 (6%)	32 (0.8%)	8.18	2.41–27.71	.0007*
Erythromycin	1 (2%)	14 (0.36%)	5.99	0.77–46.50	.0869
Bezafibrate	1 (2%)	11 (0.28%)	7.63	0.96–60.31	.0541
Ezetimibe	2 (4%)	36 (0.9%)	4.73	1.11–20.26	.**0361**
Gemfibrozil	2 (4%)	12 (0.3%)	14.29	3.11–65.71	.0006*
Pivmecillinam	2 (4%)	59 (1.5%)	2.87	0.68–12.11	.1509
Amiodarone	1 (2%)	9 (0.2%)	9.33	1.16–75.14	.**0359**

Abbreviations: CI, confidence interval; NA, not available; OR, odds ratio.

Doses are converted to simvastatin dose equivalents. Median values of continuous variables are presented with the lower quartile and the upper quartile in italics. Percentages were calculated from the total number of cases with information.

Anatomical Therapeutic Chemical (ATC) Classification codes are presented before each drug class.

Nominal significant differences (*P* < .05) are shown in bold.

a Doses are based on conversion to simvastatin dose equivalents and OR is calculated by SD 19.82. Data lacking for 7 cases.

b Numbers and proportions are based on frequency the drug was present in cases and controls, irrespective of whether the drug was suspected to be causative or not.

c Fusidic acid was not possible to analyze in the model as the treatment frequency in controls was 0.

* Statistical significant differences (*P* < .0017).

Simvastatin was the most commonly used statin in both cases and controls and the second most common was atorvastatin (Table 3). Cases were treated with higher doses than controls, in median 40 vs 20 mg/d for both simvastatin and atorvastatin. The cases’ atorvastatin doses were remarkably high; 11% were treated with the maximal dosage of 80 mg. Doses were converted to simvastatin dose equivalents (Table 2). The median simvastatin dose equivalent was 40 vs 20 mg/d in cases and controls, and a higher dose was associated with a higher risk of myopathy (OR = 1.64, 95% CI = 1.45-1.86 and *P* < .0001). This OR was calculated using the standard deviation of 19.82, which equals the OR for a 20-mg dose increase. A dose increase in 40 mg resulted in OR = 2.72, 95% CI = 2.11-3.50, ie, a 2.7 times increased risk of myopathy.

**Table 3.** Statin treatment in cases and controls.

	Cases (n = 47)^a^	Controls (n = 3871)
Statin		
Simvastatin	29 (63%)	3293 (85%)
Atorvastatin	16 (35%)	421 (11%)
Rosuvastatin	1 (2%)	43 (1%)
Fluvastatin	—	11 (0.3%)
Pravastatin	—	103 (3%)
Median dose, mg/d		
Simvastatin	*20* 40 *40*	*20* 20 *30*
Atorvastatin	*20 *40 *80*	*10 *20 *20*
Rosuvastatin	10	*10 *10 *10*
Fluvastatin	—	*40 *80 *80*
Pravastatin	—	*20 *20 *40*

Percentages were calculated from the total number of cases with information on type of statin. Median doses are presented with the lower quartile and the upper quartile in italics.

a Data on which type of statin lacking were missing for 1 case and data on dose were missing for 7 cases.

Diseases found in at least 15% of the cases were hypertension, hyperlipidemia, angina pectoris or myocardial infarction, arthrosis, diabetes, stroke (incl. transient ischemic attack), and atrial fibrillation. Hypertension, hyperlipidemia, arthrosis, and diabetes were not compared between cases and controls because of a low sensitivity in the National Patient Register.^15^ In general, markers of cardiovascular disease, such as ischemic heart disease, treatment with vitamin K antagonists, and loop diuretics, were more common in cases than in controls (Table 2). In 14 cases, other drugs in addition to statins were reported as suspected drugs (Table 2). Cyclosporine, allopurinol, ticagrelor, clindamycin and gemfibrozil were statistically significant variables among these drugs. One observation was that all cases with clindamycin treatment were coprescribed with fusidic acid. The model was not able to analyze fusidic acid as no controls had this drug. However, fusidic acid was significantly associated with myopathy using calculations in the Open Source Epidemiologic Statistics for Public Health’s module,^19^ which resulted in OR = 1002, 95% CI = 54.55-18 410, and *P* < .0001.

In the multivariate analysis, statin dose and cyclosporine were identified as statistically significant independent risk factors for myopathy (Table 4). Gemfibrozil, clindamycin, and ischemic heart disease were tested with nominal significance.

**Table 4.** Multivariate logistic regression of the association between statin myopathy and clinical factors.

Variable	OR	95% CI	*P* value
Statin dose^a^	1.54	1.32–1.80	**<.0001** *
Cyclosporine	34.10	4.43–262.45	.0007*
Gemfibrozil	12.35	2.38–64.10	.**0028**
Angina pectoris or myocardial infarction	2.35	1.16–4.74	.**0171**
Clindamycin	5.96	1.47–24.24	.**0125**
Vitamin K antagonists	2.37	0.95–5.89	.0633
Allopurinol	1.94	0.50–7.58	.0749
Ticagrelor	1.55	0.36–6.80	.5581
Loop diuretics	2.13	0.95–4.76	.0647

Abbreviations: CI, confidence interval; OR, odds ratio.

Nominal significant differences (*P* < .05) are presented in bold.

a Data lacking for 7 cases. Doses are based on conversion to simvastatin dose equivalence and OR is calculated using SD 19.82.

* Statistically significant differences (*P* < .0017).

## Discussion

In this case-control study, we found several clinical differences between statin-treated individuals who did and did not experience myopathy in association with statin treatment. Statistical analysis revealed drug-related risk factors that are consistent with results from previous studies. First, we detected high-dose statin treatment, which is one of the most commonly mentioned risk factors in the literature.^7,8,20^ The pathophysiology behind statin-associated myopathy is not fully known, but myopathy is in many cases dose dependent, ie, the risk increases with serum concentrations of statins.^8,20^ This risk must be highlighted as the current treatment guidelines focus on cardiovascular risk as a determinant of treatment intensity and recommends patients at very high risk to be treated with high-potent statins in maximal tolerable dose.^21^

Second, the interacting drugs fusidic acid, cyclosporine, and gemfibrozil were confirmed as risk factors for myopathy in this study. Most of the cases and controls were treated with simvastatin or atorvastatin. Together with lovastatin, these statins have been associated with a 4 times higher incidence of rhabdomyolysis than pravastatin and fluvastatin. The primary reason for this is that pravastatin and fluvastatin are weaker statins, but a contributing factor is that simvastatin, atorvastatin, and lovastatin are metabolized by the cytochrome P450 3A4 (CYP3A4) enzyme, which is inhibited by many commonly used drugs.^6^ Fusidic acid was the strongest risk factor in this study. Oral fusidic acid is an antibacterial agent used for the treatment of staphylococci infections in the skin and soft tissue. It is a known risk factor for myopathy when combined with statins, although it is not mentioned frequently in the literature.^22^ Fusidic acid can interact with all statins and discontinuation of the statin is recommended during treatment with fusidic acid, followed by a 7-day break before reintroducing the statin.^22,23^ Clindamycin, another antibacterial agent, does not interact with either fusidic acid or statins.^23^ The association with clindamycin in this study could be dependent on comedication with fusidic acid, as all cases treated with clindamycin also were treated with fusidic acid. How much fusidic acid contributes to or affects the variables, and vice versa, is unknown as it was not included in the multivariate model.

Cyclosporine was identified as an independent risk factor in this study and is known to interact with all statins in different degrees.^24^ As an inhibitor of the CYP3A4 enzyme and transport proteins involved in statin metabolism, a combination with statins leads to increased exposure of statins, with a maximum of a 10-fold increase for atorvastatin, pravastatin, and rosuvastatin.^24^ Statin doses should be reduced and some statins should not be taken with cyclosporine. Fluvastatin has the least increase in exposure (2-fold to 4-fold) when taken simultaneously with cyclosporine and may be an alternative when comedication is required.^23^

Gemfibrozil is another well-documented risk factor for myopathy and the association was confirmed in this study. Gemfibrozil can cause rhabdomyolysis as monotherapy, but the risk is substantially higher when coadministered with statins.^6^ It is contraindicated to prescribe gemfibrozil with simvastatin and coadministration of gemfibrozil and other statins should be avoided.^25^

Cases were treated with higher statin doses and had a higher prevalence of many markers of cardiovascular disease, ie, were more likely to be on secondary prevention than controls. One of the main indications for secondary prevention is ischemic heart disease, eg, angina pectoris and myocardial infarction, where high statin doses are recommended. Other drugs that indicate cardiovascular disease were univariately associated, but lost significance in a multivariate model that also contained statin dose, probably because of collinearity between other cardiovascular treatment and a high statin dose.

Strengths of this study are that the clinical characteristics were carefully described, and that the diagnosis myopathy was validated in every case. Using reports of myopathy sent to the MPA as a source gives this study a nationwide coverage.

## Limitations

A limitation of this study is that we were not able to retrieve detailed information from the Swedish Twin Register on the demographics and comorbidity of the controls. It is not clear how representative this group is for the statin-treated population as a whole, and selection bias cannot be excluded. The lower prevalence of ischemic heart disease in the controls might be due the way the data were collected. The National Patient Register has a low sensitivity for many common diseases, eg, diagnoses set in primary care like angina pectoris.^15^ It is therefore possible that the frequency of ischemic heart disease is underestimated in the controls. In contrast, the registry has a high sensitivity for myocardial infarction, which is a typical in-hospital diagnosis.

Another limitation of this study is the small study sample with a total number of 47 cases. One of the reasons for this is the underreporting of ADRs, with an estimation that not more than 6% of all ADRs are reported.^26^ One can assume that this mainly concerns milder forms of myopathy because muscle symptoms or CK elevations alone may be too unspecific or perhaps overlooked.

## Conclusions

Myopathy due to statin therapy is according to our results a dose-dependent ADR. The risk of commonly reported myopathic adverse reactions was increased in individuals with a high statin dose or cotreatment with the interacting drugs fusidic acid, cyclosporine, or gemfibrozil. It is therefore likely that many cases could be prevented by assessing risk factors during a simple appointment with the clinician. Furthermore, it could be of value to investigate whether an individual is predisposed to higher circulating statin concentrations, and thus a higher risk of myopathy, due to variation in the known susceptibility gene SLCO1B1.
